# Back pain, ankylosing spondylitis and social media usage; a descriptive analysis of current activity

**DOI:** 10.1007/s00296-020-04600-w

**Published:** 2020-05-19

**Authors:** Elizabeth Reilly, Raj Sengupta

**Affiliations:** 1 Department of Rheumatology, Royal National Hospital for Rheumatic Diseases, Royal United Hospital Bath NHS Foundation Trust, Combe Park, Bath, BA1 3NG UK; 2 Department of Pharmacy and Pharmacology, University of Bath, Claverton Down, Bath, BA2 7AY UK

**Keywords:** Ankylosing spondylitis, Back pain, Exercise, Social media, Internet

## Abstract

Social media usage by back pain patients is a new and developing area. Analysing patterns of this online activity offers a new way to understand our patients’ concerns and behaviour around disease. Large volume data can be evaluated on a scale not feasible through alternative methods. A cross sectional review of specific terms relating to ‘back pain’ (BP) and ‘ankylosing spondylitis’ (AS) were tracked internationally on popular websites, blogs and boards over two 3 month periods, in 2016 and 2019. Relevant co-terms were also tracked in these discussions, such as ‘exercise’, ‘medication’ and ‘doctor’. The size of the current online BP conversation is significant; there were over 100,000 mentions/month across each study period, particularly ‘low-’ BP. Discussions about AS increased threefold between 2016 and 2019. More discussions took place online at the start of the week, and in the afternoons. Pregnancy, baby and mens’ health resources were the most popular sites for BP chats. People posting about AS were mainly female (80%) and predominantly had an established diagnosis, with health forums hosting more of these discussions than for BP. Exercise was more commonly mentioned in the context of BP, whereas medications were more common in the AS conversations. Analysing online discussions about BP and AS helps to identify themes amongst patients. Some are seeking a diagnosis, support, or treatment information. Understanding the massive scale of online conversations could help clinicians adopt targeted approaches to increase patient identification, meet patient concerns better, and optimise engagement.

## Introduction

Health-related social media have seen an explosion in activity over the last few years [1, 2]. Patients are more engaged with their healthcare, and they want more detailed information [3]. This helps support the patient–clinician relationship, and optimises compliance. Self-management and exercise are invaluable elements of the management of musculoskeletal conditions and back pain (BP) of all aetiologies [4, 5], although compliance with this is much underestimated [6, 7].

Ankylosing Spondylitis (AS), a form of axial spondyloarthritis (SpA), has an estimated mean prevalence of 23.8 per 10,000 in Europe [8]. The peak age of onset is 20–30 years. Such a cohort, therefore, is very relevant for internet-based research as we know that the greatest internet use is amongst younger people [9].

The aim of this work was exploratory, to summarise trends regarding the type of individuals involved in online discussions about BP and AS, and examine the nature of the conversation at scale, including related discussions concerning exercise and medications.

## Methods

A cross-sectional review of all worldwide online mentions of BP and AS was tracked on popular websites, blogs and boards initially from August to October 2016. A repeat search was undertaken over the same months of 2019 to assess how the picture had changed in 3 years. A taxonomy of relevant terms, such as ‘back pain’ and ‘ankylosing spondylitis’, was used in appropriate permutations to maximise identification (such as ‘backpain’). These were then used in collaboration with additional relevant terms [Appendix 1, Table 1]. The most influential sites, boards and individuals (termed ‘reach’, defined by the number of posts in the dataset, multiplied by the number of followers of the user or site) were reviewed to explore who may be driving online discussions. Mentions of healthcare professionals, medications and exercise were recorded. Finally, to understand the patients’ journey, qualitative coding was used to look at randomised posts for key terms associated with disease stage and symptom duration, as well as established cases of AS.

Data collection was conducted collaboratively between the clinical team and White Swan. White Swan, a registered charity arm of Black Swan, analyses large volumes of data for predictive purposes to improve the health of society, e.g. healthcare organisations and charities [10]. Socialnaut (a social listening app, part of Black Swan’s proprietary suits of applications, NEST) and qualitative coding were used by White Swan to identify themes and trends in the discussions. It has over 80 volunteers who carry out work on behalf of the charity and consequently no funding for the project was required. Black Swan provides all technology and intellectual property to White Swan for free and data were gathered via the existing technical integrations between Black Swan and data providers. This includes the full Twitter firehose and all data available from Socialgist (the world’s largest social media analytics firm). Ethical approval was not required as the information used was in the public domain and patients were not recruited for participation. Furthermore, no patients’ identifiable information was included. All keywords were in English, although the search itself was undertaken globally. Available demographic information and the time of day or week of posts were recorded (accounting for location, and relative to Greenwich Mean Time). Results were compiled by White Swan and fed back to the clinical team.

## Results

### Back Pain

**Fig. 1:**
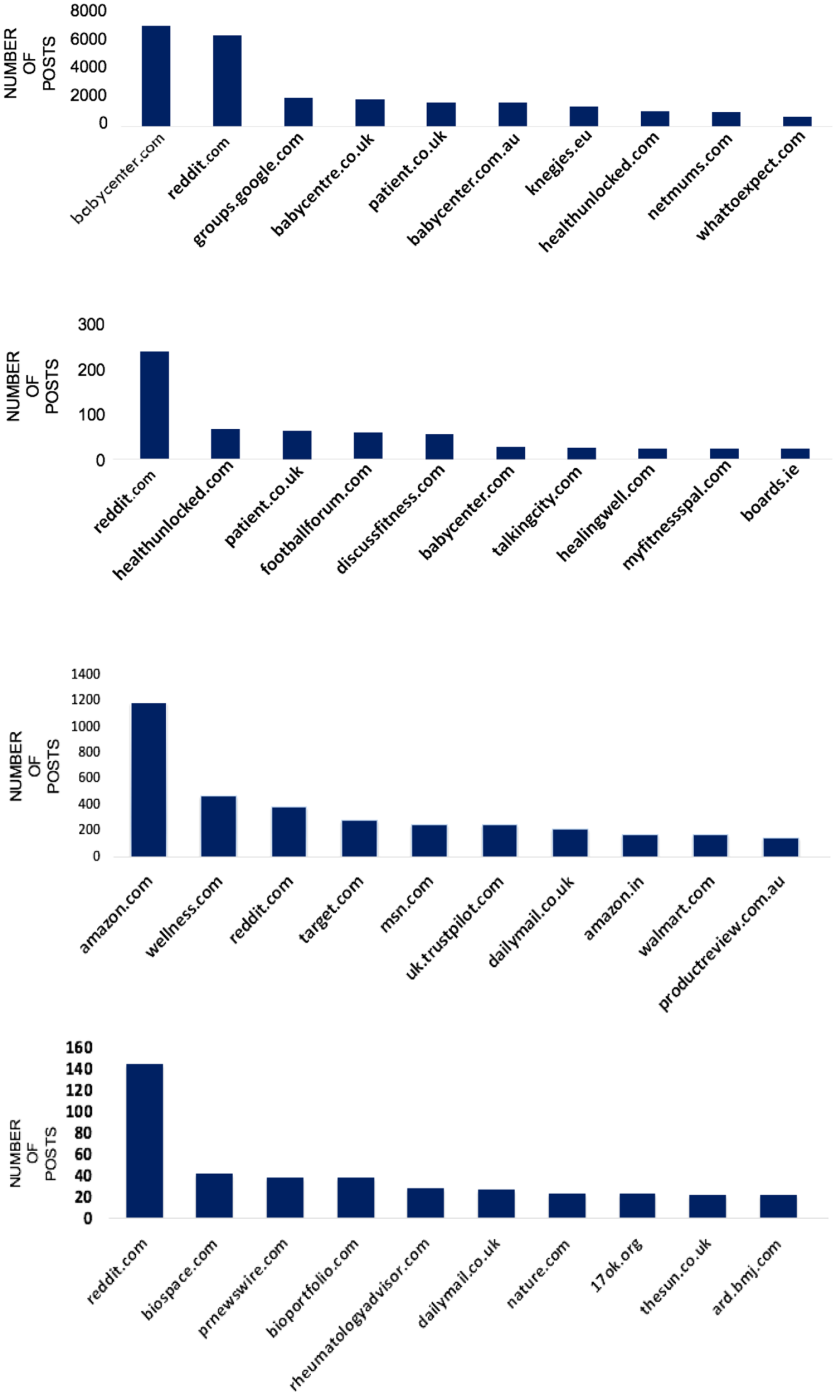
Top 10 most popular sites for conversations: 1st panel–2016: back pain, 2nd panel–2016: Ankylosing Spondylitis, 3rd panel–2019: back pain, 4th panel–2019: Ankylosing Spondylitis

The size of the current online conversation about BP is significant; there were 112,000 mentions/month of BP across the 3-month study period in 2016. This was slightly lower in 2019 at 100,000/month. ‘Low’ or ‘lower back pain’ was the most common descriptors at both time points, with a total of 62,760 mentions in 2016 and 35,204 in 2019. Metadata identified a relatively even gender split in BP discussions (52% male: 47% female). Twitter was the most frequently used site, hosting 83% of the conversations in 2016 and 93% in 2019. Online boards and forums contributed 14% of the content, and just 3% was via blogs. Patient forums (such as patient.co.uk and healthunlocked.com) also saw frequent mentions of BP (5th and 8th, respectively) (Fig. 1, top panel), as well as pregnancy or baby-related sites, (babycentre.com, netmums.com). By 2019, the most popular forum by far was Amazon, largely through product reviews mentioning BP.

**Fig. 2:**
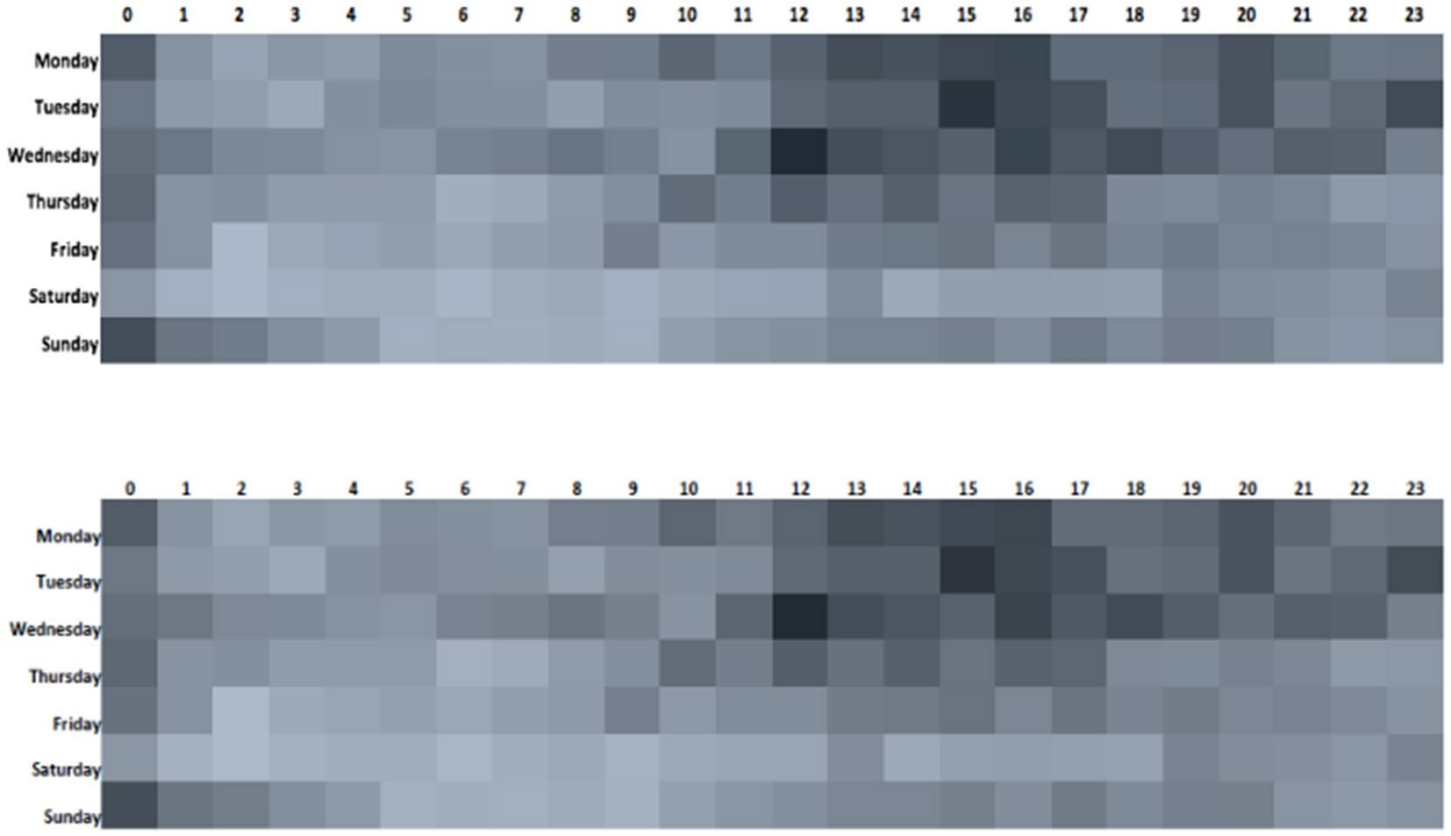
Timing of online conversations: top panel–discussions regarding back pain, lower panel–discussions regarding Ankylosing Spondylitis [depth of grey scale blocks represents amount of online activity at time points, darker = more mentions of terms in online conversations]

The top influencer on Twitter for BP in 2016 (i.e. with the greatest ‘reach’) was the user MensHealthMag, followed by a number of other similar fitness publications within the top 10. By 2019, health and fitness influencers still made up much out of the top 10. During both 2016 and 2019, mentions of BP were seen more frequently earlier in the week and between midday and 4 pm (Fig. 2, top panel). The weekends generally, Saturdays in particular, saw much less activity.

### Ankylosing spondylitis

In 2016, the international online community saw 1700 mentions/month of ‘ankylosing spondylitis’. By 2019, this had risen nearly 3 times to 4398/month. In 2019, axial spondyloarthritis (‘axSpA’) was also searched for and was only mentioned 2004 times vs 13,195 of AS. Unexpectedly, 80% of those postings about AS on boards or forums were females compared to an even split in BP discussions.

Similar to that of BP, Twitter was the commonest site for discussions (75% and 79%, 2016 and 2019, respectively). In 2016, there was a higher use of ‘through boards’ (such as Reddit) and health forums for AS (20% AS vs 14% BP) (Fig. 1, lower panel), which had reduced substantially to only 2% by 2019. The blog use of AS was similar to BP (5% vs. 3%), but baby-related boards were less common. The Spondylitis Association of America was the most popular AS-specific site in 2016, and the NASS Twitter account had the 2nd greatest reach at both time points. Many influential leaders on Twitter were individuals diagnosed with SpA or campaigners. The timing of AS posts reflected that for BP, with highest activity in the afternoons and evenings, earlier in the week (Fig. 2, lower panel).

### Exercise and medications

Exercise plays a prominent role in all discussions. At both time points, ‘exercise’ was consistently mentioned more than ‘medications’ during BP conversations (2016: ‘exercise’ 49,993 mentions vs ‘medications’ 27,306 times, 2019: 18,941 vs 4177). In comparison, ‘medications’ were more common than ‘exercise’ in AS discussions (2016: 1018 vs 525, 2019: 1382 vs 700). For BP, yoga was the commonest type of exercise (4%, 13,000 mentions), and the 7th most influential resource for AS was a yoga site, DDPYoga.

Non-steroidal anti-inflammatories were the commonest drugs mentioned in 2019, whereas in 2016, it was analgesics, such as paracetamol. Humira was the commonest biologic medication discussed in 2016 (2% of the total AS conversation), but by 2019, this was generic adalimumab and Cosentyx (Novartis). Many patients were sharing experiences of treatment.

### The patient journey

Most contributors to the online conversations (71%) had an existing diagnosis of AS. Twenty-one percent were deemed ‘pre-diagnosis’ owing to information-seeking behaviour and symptoms. They were commonly seeking or querying a diagnosis of AS by themselves, or others may be suggesting it. Some were expressing frustrations to their doctors for not taking their BP seriously. The remaining 8% gave indications that suggested a recent AS diagnosis. Patients were presenting to a variety of healthcare professionals, such as ophthalmologists, rheumatologists, GPs and some complementary therapists, such as chiropractors.

## Discussion

AS and BP patients are actively seeking information online through an array of sources with recurring topics of conversation. The conversation around AS is smaller than for BP, which is expected given that SpA represents < 5% of BP cases [11]. The AS discussion has grown substantially in 3 years, potentially through increased publicity of the disease in lay publications and the popular media and the work by patient bodies, such as NASS and Versus Arthritis. It must be appreciated though that a broader range of search terms were included in the taxonomy, albeit these made up a small additional proportion of the search. The high proportion of AS mentions by women is an unexpected result, given the 3.8:1 male to female ratio in European AS populations [5]. This very fact, however, may reduce the identification of AS in women and they may consequently be more likely to seek advice online. They could also be searching online on behalf of someone else, which could skew results. Female internet use has increased steadily over the previous two decades, with relatively similar rates of use now between genders [6, 12, 13], further making the trend seen in the AS group surprising. However, when specifically healthcare-related sites are analysed, women rate the usefulness of internet-derived health information more highly than men and also enjoy using the internet more for this purpose [14], perhaps offering an explanation. The high frequency of baby or pregnancy-focussed sites suggests that pregnancy-related BP is a common issue. The distribution of postings later in the day may coincide with increased symptoms, such as in mechanical BP. Alternatively, this could reflect a period of reprieve from symptoms, as in inflammatory BP, enabling patients to focus on spending time on the internet. The higher rate of mentions of ‘exercise’ vs ‘doctor’ for BP suggests patients understand the importance of this, particularly given the limited role of medications in mechanical BP. Given the importance of exercise in AS, one would have hoped to see more mentions, but as the options for effective medical treatment have increased in recent years, this is not unexpected. Biologic medications are newer treatments and, therefore, patients may be seeking to understand their use. Humira was reported by IMS Health in 2014 as the world’s best-selling drug (across indications) [15], so it is not surprising that this is the commonest biologic mentioned in 2016, whilst newer options, such as secukinumab have since become options for treatment. Sharing views on treatments is likely to offer security or validation of patients’ own experiences.

**Fig. 3:**
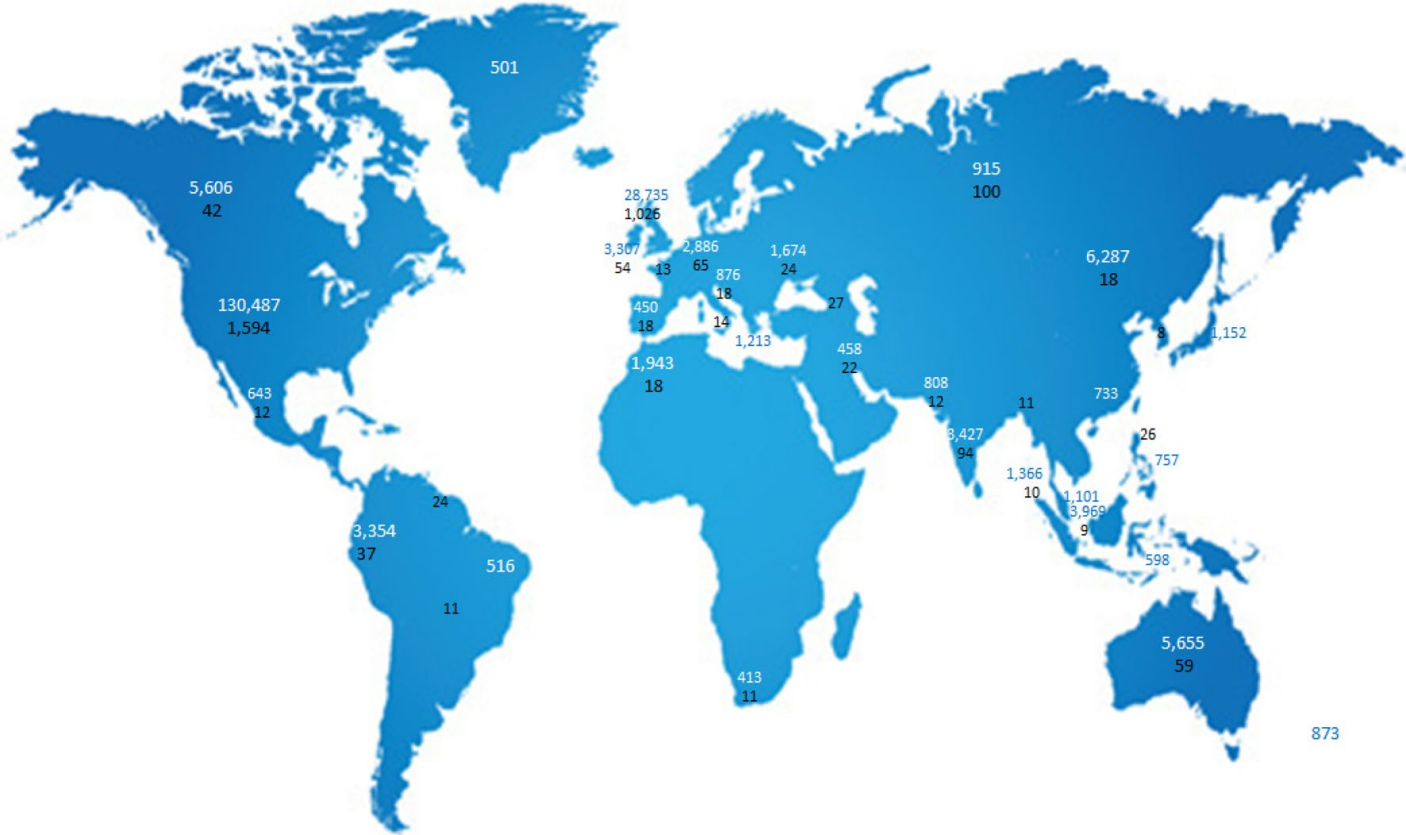
Worldwide data contribution to analysis, number of posts on topic [blue–back pain, black–AS]

There are limitations in this type of study. The level of detail is limited; for example, it is difficult to ascertain the correct usage of medical terms online by the lay public. Using Socialnaut, we have tried to be as comprehensive and inclusive as this methodology allows, but there will be some boards or sites that may not have been included. Search terms were limited to English, which may bias data towards the US and the UK sites. It was not possible to control for the potential unequal global internet provision (Fig. 3) or healthcare systems informing patients’ experiences presented online. Understanding wider online healthcare discussions may also offer alternative explanations for some of the patterns seen here. For example, does the timing of posts for BP/AS reflect that is seen in other conditions, or is it due to the diurnal variability in symptoms alone? How does the size of the BP discussion compare to that of other musculoskeletal conditions? This was outside the scope of this work. There was also limited capture of demographic information, such as age, education or other confounders. However, the magnitude of data analysed would not be possible through more traditional study methods and shows trends that can support further work.

## Conclusion

This is the first study to utilise big data analysis to describe trends of online discussions of BP and AS, and offers a rich data source relatively untouched for medical research purposes so far. The current online conversation about BP is huge, and that of AS is increasing. Through this analysis, patients appear well-informed, and particularly are seeking diagnoses, and more details on treatment options. Prompt recognition of inflammatory axial symptoms is pivotal in optimising outcomes, particularly given the diagnostic delay in AS, the longest for any rheumatological condition [16]. This sort of data could offer a novel way of flagging potential patients through their online interactions. Targeted patient information could improve patients’ understanding of their condition and highlight the importance of exercise. Regular ‘hot topic’ segments on BP could feature on commonly used sites to engage the public, and encourage presentation to healthcare professionals. The role of campaigners in these discussions emphasises on how potent the personal perspective is for patients. Collaborative work with other healthcare professionals seeing BP patients, such as chiropractors or online influencers, could improve education and facilitate an earlier medical referral. One must, however, be aware of data protection and patient confidentiality when such information is concerned, particularly as technology becomes an increasing part of day-to-day life.

## Data Availability

Data transparency: upon request. Software application or custom code: upon request from White Swan.

## Appendix 1 Search terms

See Table 1.

**Table 1:** Additional search terms from 2019 re- analysis indicated with *

Back pain and other symptoms	Back pain, pain in my back, back stiffness*, joint pain*, joint stiffness*, uveitis*
Ankylosing spondylitis	Ankylosing spondylitis, ankylosingspondylitis, AS, chronic pain, chronic pain, AS symptoms*, symptoms of AS*, sign of AS*, spine inflammation*, inflammation of the spine*
Axial SPA	Axial Spondyloarthritis*, Axial Spa*
NSAIDs	Brufen, naproxen, diclofenac, indomethacin, meloxicam, etodolac, celebrex, arcoxia, Non-steroidal anti-inflammatory drugs (NSAIDS)*
Painkillers	Paracetamol, cocodamol, codydramol, tramadol, coproxamol, morphine, painkillers*
DMARDs	Methotrexate, sulphasalazine, leflunomide, Disease-modifying drugs (DMARDs)*
Biologics	Humira, adalimumab, Enbrel, etanercept, simponi, golimumab, remicade, infliximab, cimzia, certolizumab, cosentyx, secukinumab
Exercise	Exercise, exercises, yoga, pilates, gym
